# The novel llama-human chimeric antibody has potent effect in lowering LDL-c levels in *hPCSK9* transgenic rats

**DOI:** 10.1186/s40169-020-0265-2

**Published:** 2020-02-13

**Authors:** Xinyang Li, Meiniang Wang, Xinhua Zhang, Chuxin Liu, Haitao Xiang, Mi Huang, Yingying Ma, Xiaoyan Gao, Lin Jiang, Xiaopan Liu, Bo Li, Yong Hou, Xiuqing Zhang, Shuang Yang, Naibo Yang

**Affiliations:** 1 BGI Education Center, University of Chinese Academy of Sciences, Shenzhen, 518083 China; 2 BGI-Shenzhen, Shenzhen, 518083 China; 3 China National GeneBank, BGI-Shenzhen, Shenzhen, 518120 China; 4 BGI-Hubei, BGI-Shenzhen, Wuhan, 430074 China; 5 Complete Genomics, Inc., 2904 Orchard Parkway, San Jose, CA 95134 USA

**Keywords:** PCSK9, Antibody, LDL-c, VHH-Fc, sdAb, *Pichia pastoris*

## Abstract

**Background:**

The advent of proprotein convertase subtilisin/kexin type 9 (PCSK9)–inhibiting drugs have provided an effective, but extremely expensive treatment for the management of low density lipoprotein (LDL). Our aim was to explore a cost-effective application of camelid anti-PCSK9 single domain antibodies (sdAbs), which are high variable regions of the camelid heavy chain antibodies (VHHs), as a human PCSK9 (hPCSK9) inhibitor. One female llama was immunized with hPCSK9. Screening of high affinity anti-PCSK9 VHHs was carried out based on surface plasmon resonance (SPR) technology. We reported a lysate kinetic analysis method improving the screening efficiency. To increase the serum half-life and targeting properties, the constant region fragment of the human immunoglobulin gamma sub-type 4 (IgG4 Fc) was incorporated to form a novel llama-human chimeric molecule (VHH-hFc).

**Results:**

The PCSK9 inhibiting effects of the VHH proteins were analyzed in two human liver hepatocellular cells (HepG2 and Huh7) and in the hPCSK9 transgenic Sprague–Dawley (SD) rat model. The hPCSK9 antagonistic potency of the bivalent VHH-hFc exceeded the monovalent VHH (*P *< 0.001) in hepatocarcinoma cells. Furthermore, the llama-human chimeric VHH-Fc protein had a similar reduction (~ 40%) of the LDL-c and total cholesterol when compared to the approved evolocumab in transgenic SD rat model, but with low cost. More surprisingly, the chimeric heavy chain antibodies could be persevered for 3 months at room temperature with little loss of the affinity.

**Conclusions:**

Due to the high yield and low cost of *Pichia pastoris*, lipid-lowering effect and strong stability, the llama-human chimeric antibody (VHH-Fc) offers a potent therapeutic candidate for the control of the serum lipid level.

## Background

High levels of low density lipoprotein cholesterol (LDL-c) can increase the risk of cardiovascular diseases (CVD) which are associated with devastating sequelae like stroke and hemiplegia [6]. Statins, also known as β-hydroxy-β-methylglutaryl-CoA reductase inhibitors, can reduce LDL-c by ~ 25% and lower CVD risk by ~ 20%. Unfortunately, statin-resistant patients often have to opt for treatment disruption [27]. High risk patients, often with familial hypercholesterolemia, are unable to reach acceptable LDL-c levels (~ 100 mg/dL) relying on statins alone [6]. Studies have also reported that the use of statins can increase the progression of vascular calcification and skeletal muscle injury and the levels of the proprotein convertase subtilisin/kexin type 9 (PCSK9) [17, 21, 26, 31]. Due to this, the need for other molecules that can reduce LDL-c levels are required.

In 2003, human genetics studies reported the ninth and last member of the PCSK family [28]. It demonstrated that the *PCSK9* gene was the third locus of autosomal dominant hypercholesterolemia, following the *LDL receptor* (*LDLR*) and *apolipoprotein B* genes [1]. PCSK9 protein plays a vital role in cholesterol homeostasis by binding to the LDLR. High level PCSK9 competitively binds LDLR with LDL-c, which would cause disorder of LDL-c metabolism (Additional file 1: Fig. S1). PCSK9 regulates plasma LDL-c levels by regulating the degradation of LDLR [20]. It is a member of the proteinase K subfamily of subtilisin-related serine endoproteases. Like other members of this family, the PCSK9 protein has a signal sequence, followed by a prodomain, and a catalytic domain [16] (Additional file 2: Fig. S2). The crystal structure of PCSK9 could be acquired by the accession number: PDB 2QTW at the website (https://www.ebi.ac.uk/pdbe/entry/pdb/2QTW).

Apart from statins, anti-PCSK9 monoclonal antibody is also used for lowering LDL-c. Evolocumab and alirocumab are two PCSK9 inhibitors that were approved by US FDA in 2015 [25]. However, not all patients can afford these expensive drugs (≥ 5850$ every year), especially in less-developed areas [4]. Camelid single domain antibody (sdAb) is essentially the high variable region (VHH) of the heavy chain antibody (HcAb). It is also known as nanobody because of its size at the nanometer scale and may be an attractive alternative to the immunoglobulin gamma (IgG). It has many merits over IgG. For instance, its molecular weight is only ~ 15 kDa and it can penetrate the blood brain barrier. And it can be produced with low cost and high yield and stability in not only yeast, but bacteria as well [2, 19]. Recently, the first nanobody drug, Caplacizumab, was approved by the European Commission. It is a bivalent VHH designed for the treatment of thrombotic thrombocytopenic purpura and thrombosis [24]. In this study, the monovalent represents the single VHH. The bivalent VHH represents double VHH dimerized by VHH-Fc. Fc represents the constant region fragment of the human immunoglobulin gamma sub-type 4.

**Fig. 1:**
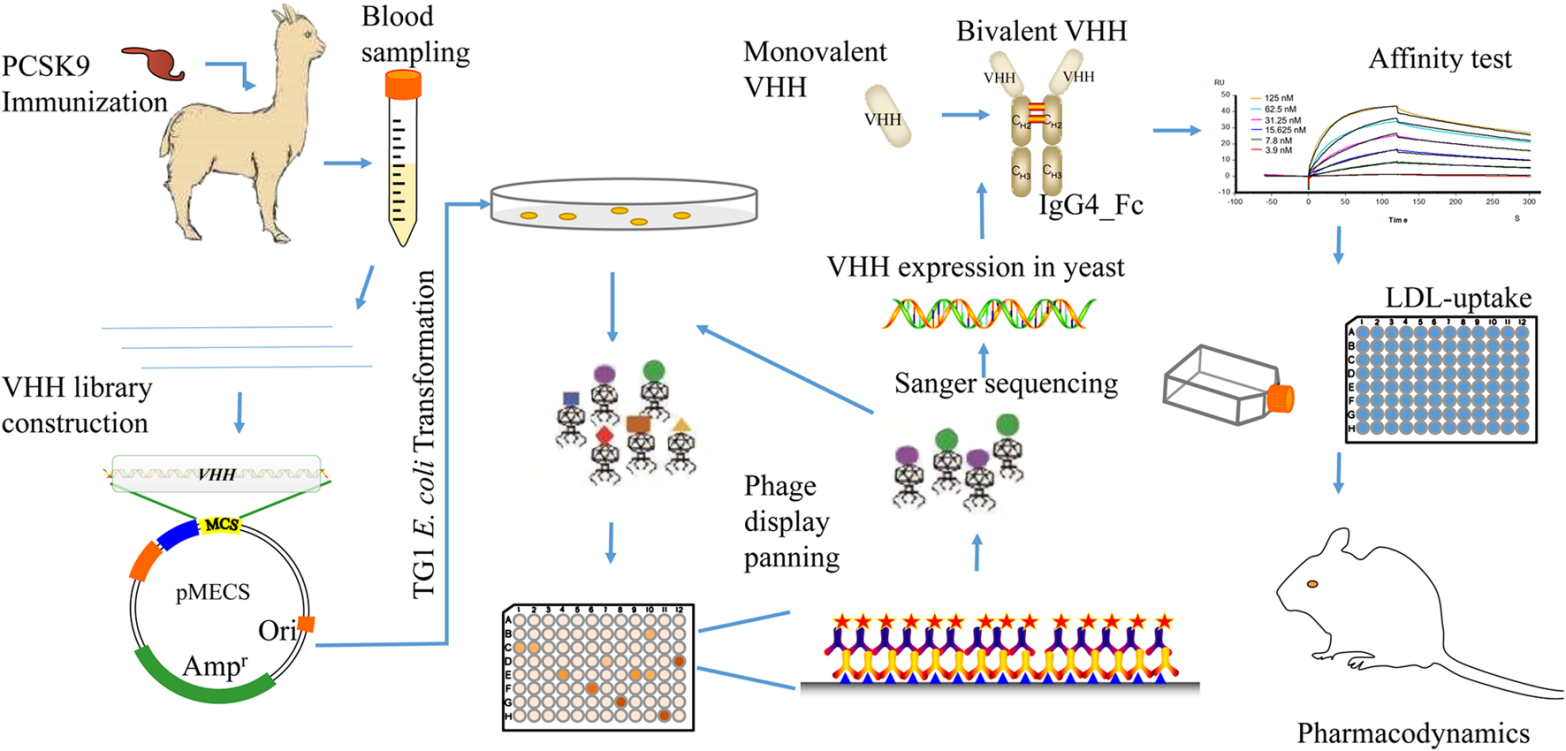
Schematic diagram of the whole experimental workflow. The whole experimental workflow consists of the PCSK9 immunization, blood sampling, VHH library construction, *E. coli* transformation, phage-display panning, Sanger sequencing, VHHs protein expression, bivalent VHH design, affinity test, LDL-uptake, and pharmacodynamics assay

In our study, we aim to explore the therapeutic application of camelid anti-PCSK9 VHH proteins on lipid-lowering effect. Following llama immunization of full-length hPCSK9, we screened for sdAbs that have high affinity to hPCSK9. The experimental flow graph was shown in the Fig. 1. The llama-human chimeric antibody we designed, could lower LDL-c by inhibiting PCSK9 in hepatoma carcinoma cells and hyperlipidemic animal models.

## Results

### Immunization and library construction

After four rounds of human PCSK9 (hPCSK9) antigen stimulations, the serological enzyme-linked immunosorbent assay (ELISA) was performed with coating (+) and no coating (−) of 100 ng hPCSK9 antigen/well. The results in Additional file 3: Fig. S3 showed the titer of the polyclonal antibody specific to the hPCSK9 was 1: 1,562,500 [the OD450 ratio of post-immune serum/pre-immune serum ≥ 2.1 is recognized serology positive (#)]. The antibody library was then constructed and the library size was measured by counting colonies after the competent TG1 *E. coli* transformation and tenfold dilution. The hPCSK9 immune library size was predicted to be about 1 × 10^10^.

### Phage display and kinetics screening

**Fig. 2:**
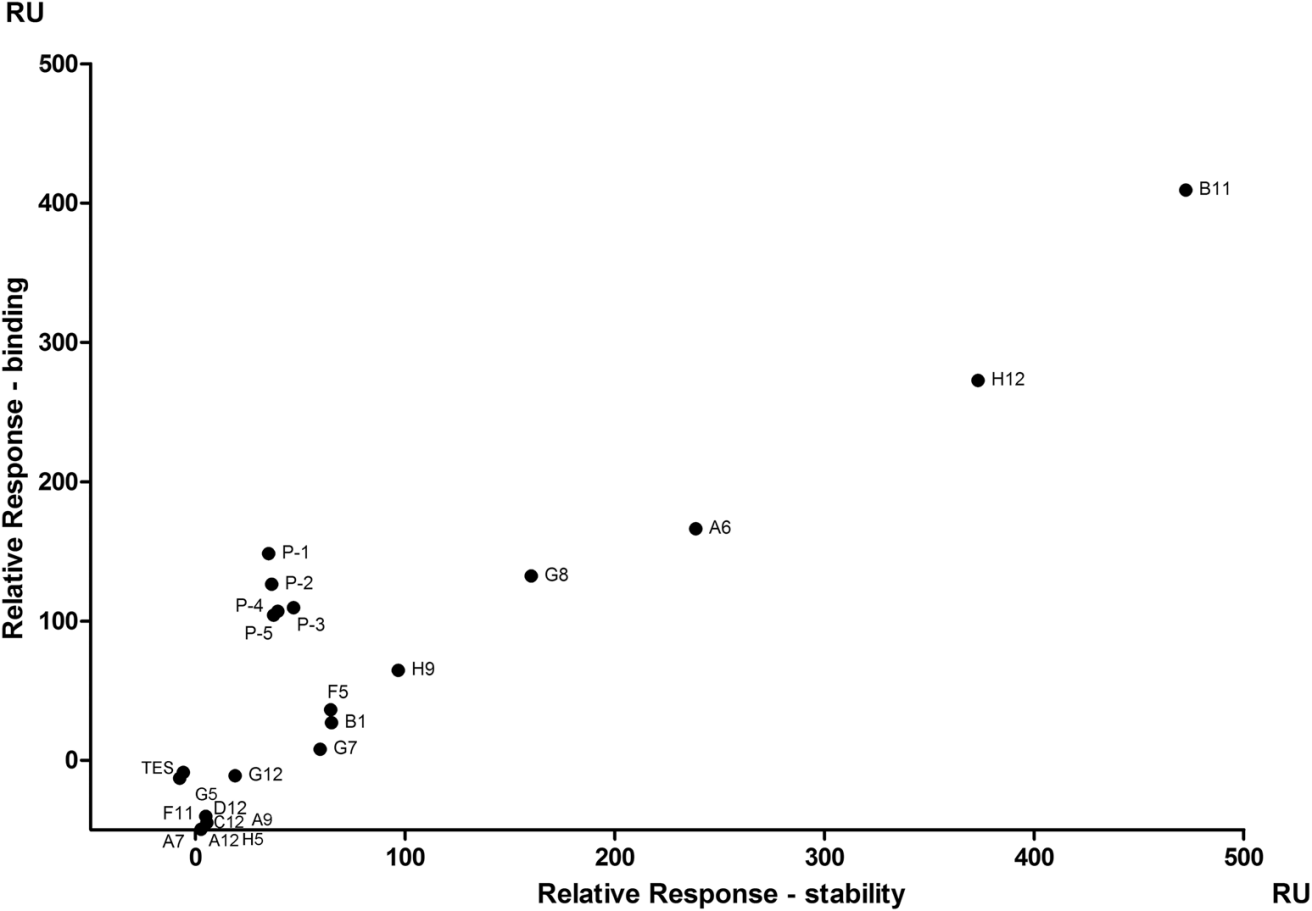
The scatter plot of the lysate kinetics screening. The scatter plot represents the results of the lysate kinetics screening. The horizontal axis represents the relative response unit of the stability. The vertical axis represents the relative response unit of the binding. Each dot represents one sample with the tag name (e.g. B11, H12, P-1 and so on). ‘TES’ represents the 55% TES buffer (0.2 M Tris–HCl pH 8.0, 0.5 mM EDTA, 0.5 M sucrose), which was system buffer and taken as the blank sample

After the fourth bio-panning, the phages produced by 22 individual clones were selected. Their supernatants of TES lysate were tested for their binding abilities to the hPCSK9. As shown in Fig. 2, the scatter plot of the relative stability and binding values illustrate the kinetics screening result, among which four sdAbs (VHH-B11, H12, A6 and G8) had apparent high binding and stability values (> 100 RU). Specifically, VHH-B11 has the highest binding (409.3 RU) and stability (472.3 RU) values. VHH-H12 has the second highest binding (272.5 RU) and stability (373.3 RU) values. VHH-A6 binding and stability values are 166.4 RU and 238.6 RU. The values of VHH-G8 are 132.5 RU and 160.1 RU. It suggests that VHH-B11 is probably the best candidate with the highest affinity for hPCSK9. Sanger sequencing verified that they had different VHH sequences (shown in the Additional file 4: Table S1). They have different IGHV and IGHJ genes usages when aligned to the *Vicugna pacos* germline gene by IMGT/V-QUEST (http://www.imgt.org/IMGT_vquest/input).

### VHH expression and protein-base assays

**Fig. 3:**
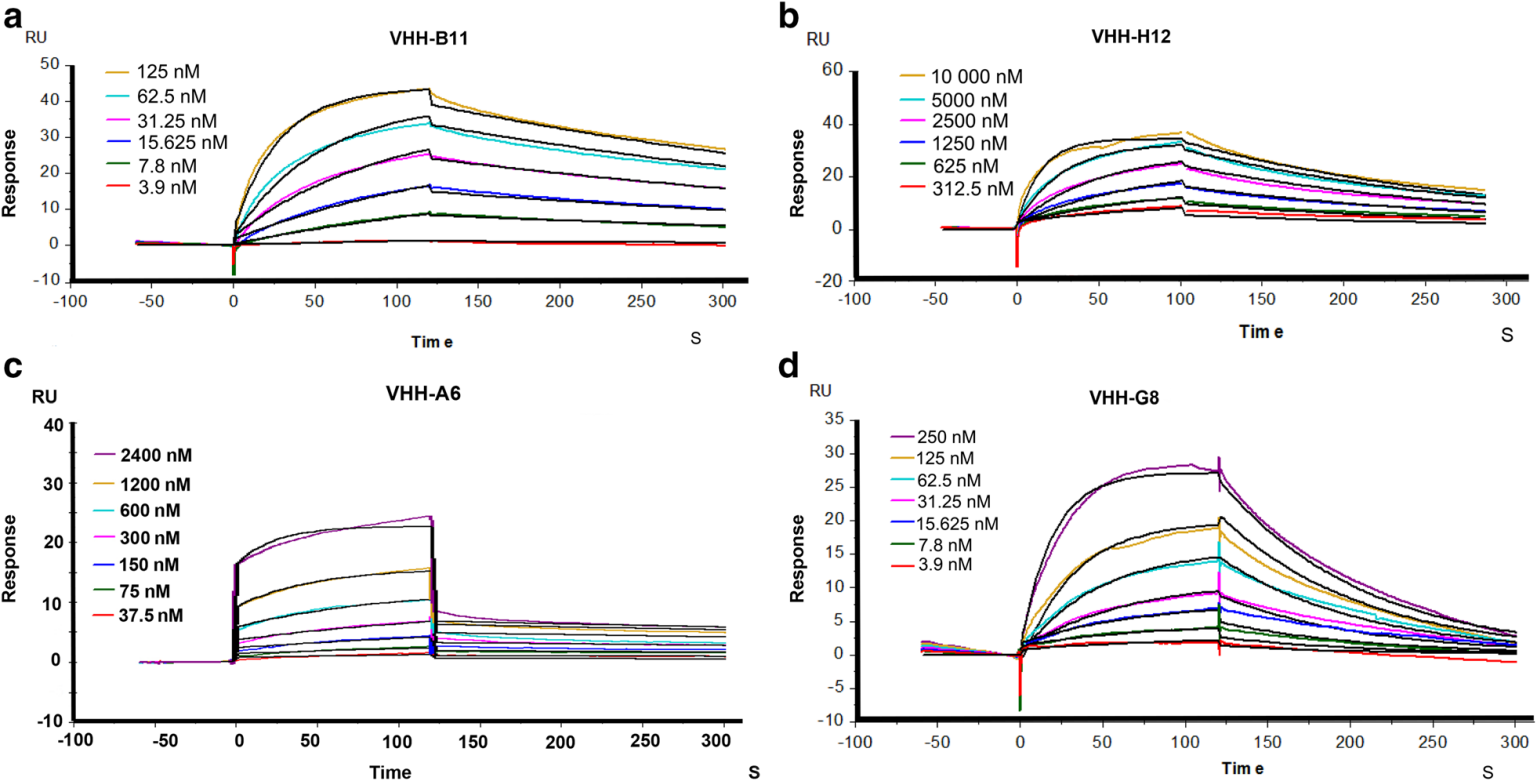
The affinity test for the four VHHs/hPCSK9 interaction. A 6 or 7-points, double fold dilution of these VHH-based proteins were injected to bind with the hPCSK9. Each colored line represents one concentration of the VHH proteins. The black lines represent the automatic fitting curves by the built-in evaluation software. The horizontal axis represents the timeline, and the vertical axis represents the relative response unit of the VHH/hPCSK9 interaction. The VHH protein injection time point was automatically set as 0 s by the built-in evaluation software

After screening, expression was induced for these four recombinant *E. coli* by 0.1 M isopropyl-β-d-thiogalactoside (IPTG). The affinity of four VHH proteins was measured by the surface plasmon resonance (SPR), as shown in the Fig. 3a–d and Table 1. We could see that each VHH had its own performance. SPR test yielded the best affinity for the B11/hPCSK9 interaction at 8.688 nM. H12/hPCSK9 interaction also had a good affinity at 703.7 nM. A6 and G8 showed a quick dissociation after binding to the hPCSK9, with the affinity at ~ 29 nM and ~ 73 nM. Therefore, B11 was selected for in-depth study.

**Table 1:** The affinities of the antibodies with the hPCSK9

Ab ID	k_on_ (1/Ms)	k_off_ (1/s)	R_max_ (RU)	Chi^2^ (RU^2^)	K_D_ (nM)
VHH-B11	2.706 × 10^5^	2.351 × 10^−3^	42.4	1.17	8.688
VHH-H12	0.7441 × 10^4^	5.236 × 10^−3^	34.94	3.53	703.7
VHH-A6	1.543 × 10^4^	4.468 × 10^−4^	15.40	0.334	28.97
VHH-G8	1.625 × 10^5^	11.44 × 10^−3^	33.64	0.813	73.05
B11-Fc (25 °C)	1.872 × 10^6^	1.288 × 10^−3^	24.71	0.693	0.6879
B11-Fc (1st week)	1.393 × 10^5^	6.181 × 10^−5^	464.6	30.1	0.4438
B11-Fc (4th week)	1.362 × 10^5^	9.116 × 10^−5^	457.4	25.3	0.6693
B11-Fc (7th week)	1.352 × 10^5^	6.983 × 10^−5^	234.8	6.82	0.5166
B11-Fc (10th week)	1.304 × 10^5^	6.523 × 10^−5^	161.9	3.22	0.5002
B11-Fc (13th week)	1.221 × 10^5^	8.148 × 10^−5^	119.5	1.82	0.6673
B11-Fc (40 °C)	4.260 × 10^5^	5.233 × 10^−5^	325.1	24.3	0.1228

Ab ID represents the name of each antibody. k_on_ (1/Ms) and k_off_ (1/s) values, respectively represent the binding and dissociation levels of the VHH interacted with the hPCSK9. K_D_ (M) was the measured affinity value acquired by the k_off_ value divided by the k_on_ value. R_max_ (RU) represents the maximum binding response unit. Chi^2^ (RU^2^) represents the curve-fitting accuracy, generally ≤ 1/10 R_max_ (RU). 25 °C/40 °C represents the reaction temperatures of the affinity determination assay by SPR. B11-Fc (xx week) represents their affinities which were detected at the preservation end of the 1st, 4th, 7th, 10th, and 13th weeks

**Fig. 4:**
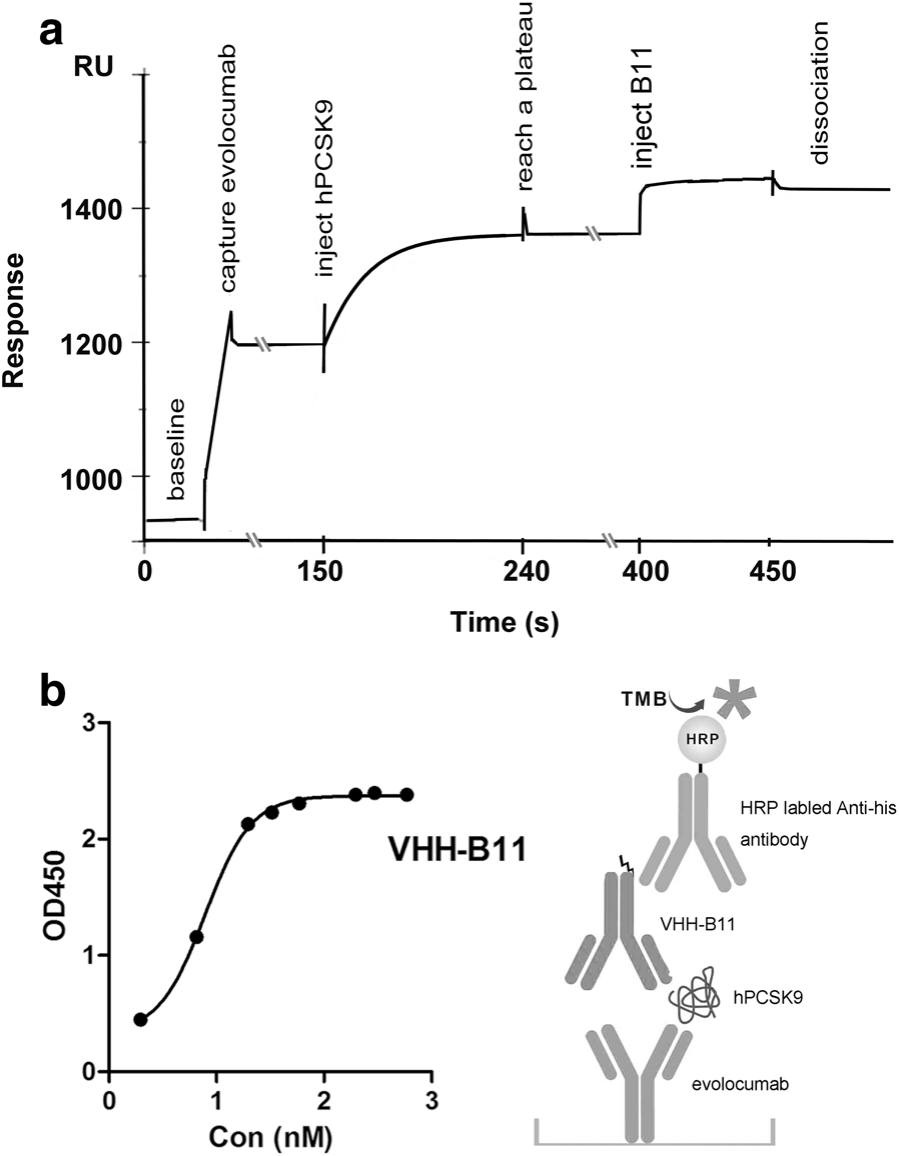
The epitope binning assay by SPR and ELISA. **a** The epitope binning assay of the VHH-B11 was performed against the approved evolocumab by SPR (Protein A chip). The horizontal axis represents the timeline. The vertical axis represents the relative response unit. The curve consists of four processes: baseline; capture evolocumab; injecting PCSK9 (binding and dissociation); injecting VHH-B11 (binding and dissociation). **b** The dual antibody sandwich ELISA method between VHH-B11 and the approved evolocumab (coated on the plate, as shown in the schematic diagram). The horizontal axis represents different dilution concentrations of the VHH-B11. The vertical axis represents the OD450 value

The epitope binning assay of the VHH-B11 was performed against the approved evolocumab by SPR. The curve in Fig. 4a consists of four processes: baseline; capture evolocumab; injecting PCSK9 (binding and dissociation); injecting VHH-B11 (binding and dissociation). It shows that after capturing evolocumab and injecting PCSK9, the evolocumab-specific epitopes on hPCSK9 were all occupied by itself and the curve reached a plateau (~ 1350RU, a stable level). Then injecting VHH-B11 still promoted to a new level (~ 1420RU). It suggests that VHH-B11 has different binding hPCSK9 epitopes with the evolocumab. From Fig. 4b, the dual antibody sandwich ELISA result also proves this fact and the method is illustrated on the bottom-right.

### Bivalency design and affinity test

**Fig. 5:**
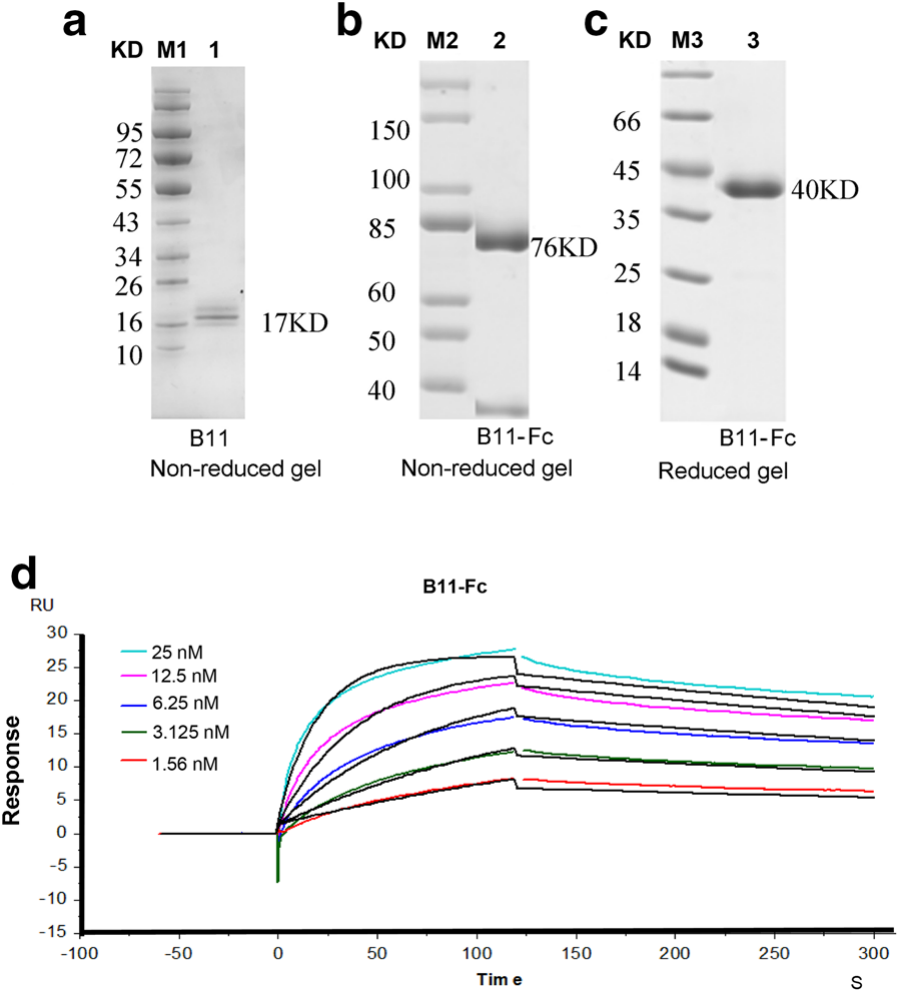
The SDS-PAGE gel and the affinity tests. **a**–**c** the SDS-PAGE gel of the VHH-B11, and the bivalency B11-Fc. M1, M2 and M3: the protein markers (Fermentas, USA). Lane1: VHH-B11, 17 kDa Lane2: B11-Fc, 76 kDa non-reduced gel; Lane3: B11-Fc, 40 kDa reduced gel. **d** The affinity test of the bivalency B11-Fc. Each colored line represents one antibody concentration. The black lines represent the automatic fitting curves by the built-in evaluation software. The binding and dissociation time was set at 180 s and 240 s, respectively, and the protein injection time point was automatically set as 0 s by the built-in evaluation software

To increase the molecular weight, stability and half-life period of the VHH binder, the bivalency structure was designed. The human IgG4 Fc was fused to the VHH-B11 to form the llama-human chimeric bivalency (B11-Fc). It was expressed in *Pichia pastoris* X33. From the Fig. 5a–c, electrophoresis results show that the B11 (17 kDa) and B11-Fc (40 kDa reduced and76 kDa non-reduced) were correctly expressed.

The affinity was also tested by the SPR (Fig. 5d). The B11-Fc proteins had high affinity with the hPCSK9 antigen at 0.6879 nM (Table 1). The engineered bivalent B11-Fc was up to ~ 12 times more potent than VHH-B11 (8.688 nM) based on the affinity.

### LDL-uptake test

**Fig. 6:**
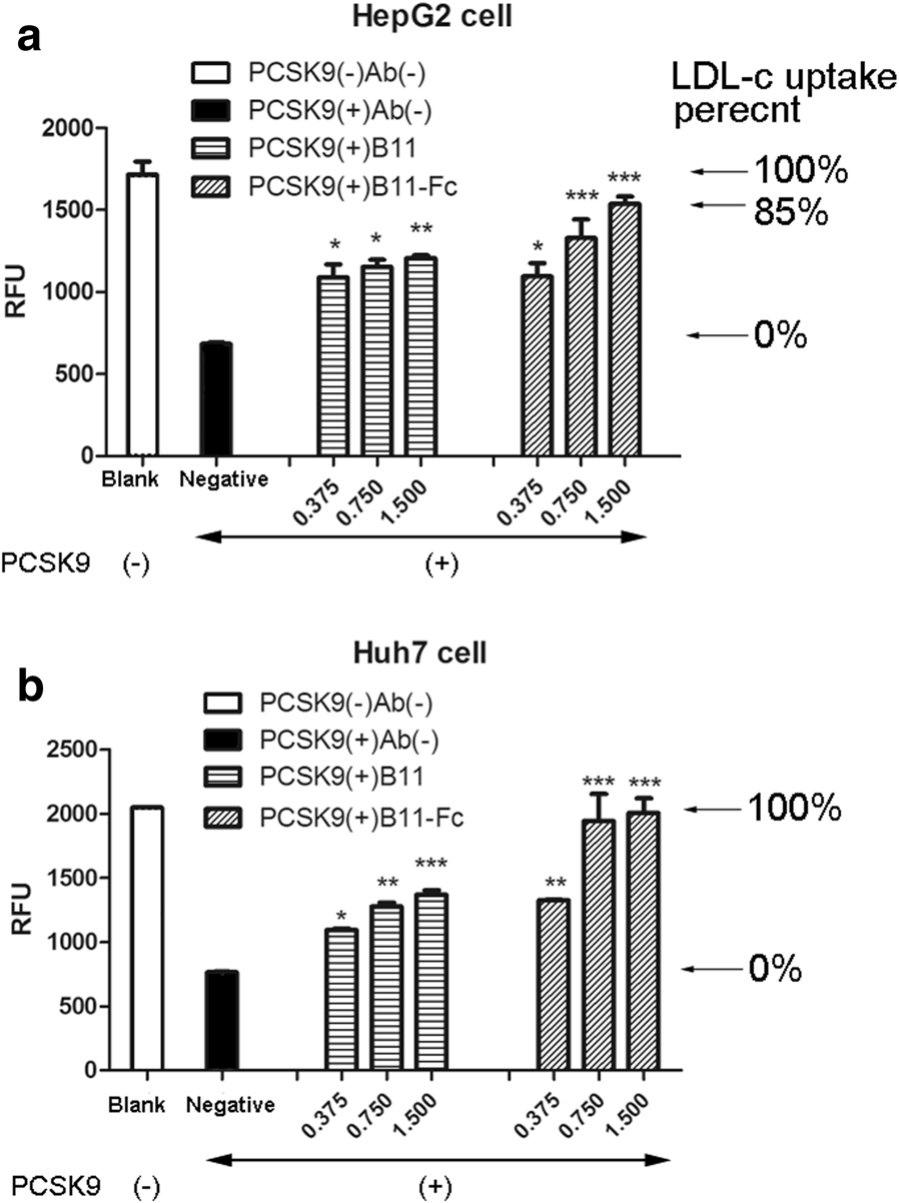
The LDL-uptake test in HepG2 (**a**) and Huh7 (**b**) cell models. The horizontal axis represents four different groups. The vertical axis represents the relative fluorescence unit (RFU) of the intracellular LDL, which reflects the levels of the LDL-c uptake and functional LDLR at the cell surface. The left two columns were respectively taken as the blank (full white) and negative (full black) control groups. The right six columns represent the other two groups of the monovalent VHH-B11, and the bivalency B11-Fc respectively in three doses (0.375, 0.750 and 1.500 μM). LDL-c uptake percent was given on the right of the histogram (←). ‘(+)’ and ‘(−)’refers to the addition and absence of hPCSK9 or VHH derivative antibodies, respectively. *P* < 0.05 was considered as statistically significant, compared with the negative control group (*P < 0.05, **P < 0.01, ***P < 0.001)

LDL binds to the LDLR on the cell surface and delivers cholesterol via receptor-mediated endocytosis. The PCSK9 competitively bind the LDLR. The labeled LDL complexes (LDL-BODIPY) were used for studying the Abs’ inhibitory effect on hPCSK9. The relative fluorescence unit (RFU) represents the LDL-c endocytosis. The stronger inhibitory effect the VHH-based Ab has, the stronger fluorescence signal it produces. As seen in the left of Fig. 6a, b, the addition of hPCSK9 alone could lead to a ~ 60% fluorescence decrease in both human hepatocellular cells (HepG2 and Huh7), which is consistent with previous reports [9, 34]. Each antibody showed different hPCSK9-inhibiting effects in a concentration-dependent pattern. The formula for the inhibition rate is as follows:

$${\text{The}}\;{\text{PCSK9}}\;{\text{inhibition}}\;{\text{rate}}\;(\% ) = {{{\text{RFU}}\left[ {\left( {{\text{no}}\;{\text{PCSK9}}\;{\text{group}}} \right) - \left( {{\text{adding}}\;{\text{Ab}}\;{\text{group}}} \right)} \right]} \mathord{\left/ {\vphantom {{{\text{RFU}}\left[ {\left( {{\text{no}}\;{\text{PCSK9}}\;{\text{group}}} \right) - \left( {{\text{adding}}\;{\text{Ab}}\;{\text{group}}} \right)} \right]} {{\text{RFU}}\left[ {\left( {{\text{no}}\;{\text{PCSK9}}\;{\text{group}}} \right) - \left( {{\text{PCSK9}}\;{\text{alone}}\;{\text{group}}} \right)} \right]}}} \right. \kern-0pt} {{\text{RFU}}\left[ {\left( {{\text{no}}\;{\text{PCSK9}}\;{\text{group}}} \right) - \left( {{\text{PCSK9}}\;{\text{alone}}\;{\text{group}}} \right)} \right]}}*100\% .$$

As expected, the bivalency B11-Fc, showed a stronger inhibiting appearance than the monovalency VHH-B11 in both HepG2 and Huh7 cells. In HepG2 cells, on addition of 1.5 μM bivalency antibody, the hPCSK9-inhibiting rate reached ~ 85%. However, only 0.75 μM bivalency antibody (B11-Fc) could completely ~ 100% inhibit the hPCSK9 in Huh7 cells.

### Pharmacodynamics

**Fig. 7:**
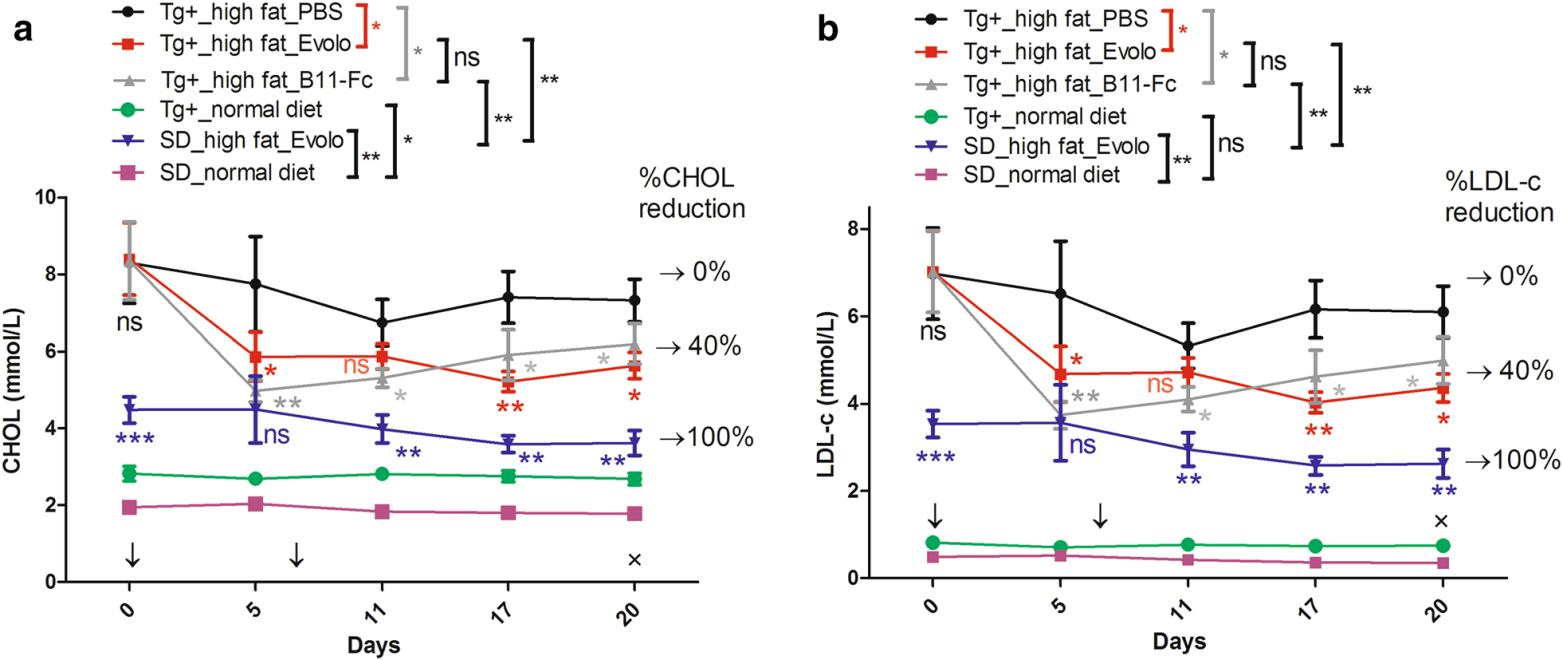
The pharmacodynamics assay in the *hPCSK9* transgenic rat model. The horizontal axis represents the timeline (days post the first treatment). The vertical axis represents the CHOL (A), LDL-c (B) levels (mmol/L) and their percent change (%) was given on the right (←). ‘SD’ was the normal Sprague Dawley rat. ‘Tg+’ represents the *hPCSK9* transgenic SD rat; ‘high fat’ refers to the feeding of the high fat diet to the rat. ‘Evolo’ represents the rat treated with the evolocumab. ‘PBS’ represents the rat injected with the PBS buffer. ‘↓’ represents the two treatments on day 0 and day 7. ‘×’ represents the sacrifice of the animals on day 20. *P* < 0.05 was considered as statistically significant, compared with the PBS group (*P < 0.05, **P < 0.01; ns: not significant)

To further verify the effect of the B11-Fc on lowering serum lipids in vivo, the *hPCSK9* transgenic (Tg+) rat model was produced and induced by a high-fat diet. Before the formal experiment, we did a pre-experiment to probe an optimal dosage (20 mg/kg) according to the previous study [33]. In the formal experiment, the dual injections were on day 0 and day 7. Rats were bled on days 0, 5, 11, 17 and 20 (then sacrificed). As seen in Fig. 7, compared with the SD_normal diet rats, high fat diet elicited a significant rise in the two types of the serum lipid for the SD_high fat_Evolo group rats (purple vs blue lines, *P* < 0.01) in the all 20 days. As expected, injecting evolocumab had no effect for the normal SD rats fed with high fat diet, so their CHOL and LDL-c levels on days 20 were taken as 100% reduction. On day 0, the three Tg^+^ groups rats fed with the high fat diet had a higher CHOL (~ 8.2 mmol/L) and LDL-c (~ 7.2 mmol/L) levels than that of the other three control groups (*P* < 0.001). On day 5, the first injecting of B11-Fc protein elicited a downward trend (~ 3.2 mmol/L CHOL or LDL-c, gray lines) for the Tg^+^_high fat_B11-Fc group. For first injecting the evolocumab, there was also a downward trend (~ 2.4 mmol/L CHOL or LDL-c, red lines) in the Tg^+^_high fat_Evolo group rats. Compared with the serum lipid level of the SD_high fat_Evolo group (blue lines), they were such declines that there was no significant difference between each group (*P* > 0.05, ns). Between day 5 and day 20, for the Tg+_high fat_Evolo group rats (red lines), two type of the serum lipid remain at the similar levels. As to the Tg+_high fat_B11-Fc group (gray lines), there were slight upward trends. This may be the results of the effects of the shorter half-period of the B11-Fc than the whole IgG. However, on day 20, the B11-Fc group had similar CHOL level with that of the Tg^+^_high fat_Evolo group (~ 6.08 vs ~ 5.83 mmol/L, ns). Besides, it was found that the LDL-c situations were similar (B11-Fc vs evolocumab group: 4.9 vs 4.5 mmol/L, ns). We also observed that Tg^+^ rats injected with B11-Fc and evolocumab had significantly lower CHOL levels (~ 6.08 and ~ 5.83 mmol/L) than that of the Tg^+^_high fat_PBS group (~ 7.5 mmol/L) (*P* < 0.05). As to LDL-c, the Tg^+^ rats injected with B11-Fc and evolocumab had significantly lower levels (~ 4.9 and ~ 4.5 mmol/L) than that of the Tg^+^_high fat_PBS group (~ 6.01 mmol/L) (*P* < 0.05). On the whole, compared with that of the SD_high fat_Evolo group (100% reduction), the B11-Fc elicited a considerable ~ 40% reduction (gray line vs blue line, *P* < 0.05) for the Tg+_high diet_B11-Fc group rats, whether on CHOL levels or on LDL-c levels. The situation of the Tg+_high diet_Evolo group was similar. The evolocumab also elicited a ~ 45% reduction on serum lipid levels, but the differences between the Tg+_high fat_B11-Fc and Evolo groups was not statistically significant (gray line vs red line, *P* > 0.05, ns).

### Stability evaluation

Furthermore, we evaluated the B11-Fc’s storage stability in 1 × PBS buffer at room temperature (~ 25 °C) by the affinity determination (Table 1 and Additional file 5: Fig. S4). From Table 1 and Additional file 5: Fig. S4A–E, at the ends of 1st, 4th, 7th, 10th, and 13th weeks, the affinities of the B11-Fc with the hPCSK9 were all detected by SPR. It is observed that they had similar affinity curves and were all at 0.1 nM scale. Even after 13 weeks’ storage, the B11-Fc still had 0.6673 nM affinity for the interaction with the hPCSK9. It suggested that the B11-Fc antibody has strong storage stability.

The temperature range of the human body is 35–39 ℃. We also evaluated the effect of reaction temperature on affinity. Compared with the affinity 0.6879 nM at 25 °C (Fig. 5),the affinity at 37 °C was also at 0.1 nM scale (data not shown). More intriguing is even at 40 °C reaction temperatures, the B11-Fc still had 0.1228 nM affinity for the interaction with the hPCSK9 antigen (as shown in Table 1 and Additional file 5: Fig. S4F). Their affinities difference is no more than six folds. All the results suggested that the B11-Fc antibody has strong stability. It would greatly facilitate the storage and transportation at room temperature.

## Discussion

In this study, a large phage immune library, about 10^10^, was constructed after immunizing a llama with the hPCSK9. From this VHH pool, one high specific sdAb (VHH-B11) with high affinity for hPCSK9 interaction was screened. Furthermore, a VHH-Fc bivalent antibody was designed which exhibited inhibition of the human PCSK9 in hepatocarcinoma cell line as well as in transgenic rats.

In the mAb screening stage, it always produced many candidates with the almost the same OD450 values. It would be very time consuming that all the VHH proteins were purified and affinity was determined. To improve the screening efficiency, based on the traditional phage display, kinetics analysis by the SPR was carried out by direct using the supernatants. This analysis gives a clear visualization between the binding and stability values using a scatter plot (Fig. 2). The VHH clone with high binding level represents a good antibody candidate [35]. And then we only need to express and purify the antibodies with high binding values. Multiple concentrations of the purified antibodies are required to obtain accurate affinity (Fig. 3).

Previous studies reported that sdAb has a short half-life period in vivo [5, 7, 15]. To improve it, we designed the Fc-fused bivalent (B11-Fc) [14], which had ~ 12 times higher affinity and better LDLR degradation inhibiting ratios than monovalent VHH (Table 1). At first the tandem bivalent (e.g. VHH–VHH), as a frequent bivalent format, is taken into account. It can also lead to an increase in affinity. However, previous study, showed the Fc-fused VHH protein (75 kDa) has a longer half-life period (~ 15 days) than that (~ 60 min) of the tandem bivalent (35 kDa), also including monovalent (~ 30 min) [7, 27]. It suggests that it would increase biological half-life period by fusion with the Fc. The results of the pharmacodynamics in Fig. 7 seems to suggests that the antibody with long half-life period has durable effect. Besides, for the VHH-Fc protein, it could further lengthen the half-life period by some amino acid mutations in the Fc region [29]. Another alternative would be to fuse it with the anti-human serum albumin VHH [30]. The Fc region also plays an important role in antibody dependent cell-mediated cytotoxicity, further enhancing B11-Fc properties in comparison to the non-IgG like formats [22]. All these indicates that Fc fusion is a simple and effective pathway for VHH engineering modification.

Furthermore, camelidae heavy chain antibody (B11-Fc)’s molecular weight is ~ 76 kDa and the productivity is better than the whole IgG (150 kDa) [3, 8, 11]. It can be expressed in yeast with low cost, but with high yield (2 to 5 g/L) after five days’ fermentation. In contrast, it needs to take 10–15 days for the mammalian cell expression system to reach this expression level [10, 12]. It is estimated that the cost of yeast expressed proteins is 23 €/g lower than that of mammalian cells (96 vs 119 €/g antibody Fab protein) [18].

As to the LDL-uptake assay, similar studies have shown that evolocumab exhibited a similar > 80% inhibition between 0.1 and 0.3 μM, and a 100% inhibition at 1 μM in HepG2 cells [34]. However, in this study, 0.75 μM bivalency antibody (B11-Fc) could completely ~ 100% inhibit the hPCSK9 in Huh7 cells. It may be caused by that the different sensitivities to the protein drugs in both cell types. On the whole, the Fc-fused VHH protein (B11-Fc) had a good pharmaceutical effect on two human hepatoma cell lines.

It is worthy to note that in pharmacodynamics assay, the Tg^+^_high fat_PBS group rats had a serum lipid decrease on day 11. This may be due to by some instability of the high fat fed rat model on these days. However, the serum lipid level gradually recovered in the next 9 days. Besides, this phenomenon was not observed in the other control groups.

In previous reports, the researchers discovered of the cryptic peptide-binding sites on PCSK9 and they can be used for the design of small molecular antagonists [23, 36]. In most instances, small molecular drugs have very short half-life period and major side effects. However, it is important that these novel binding sites can also be used for the antibody discovery.

Besides, the B11-Fc has strong affinity stability at room temperature (Table 1 and Additional file 3: Fig. S3). However, the approved drugs must store and transport at low temperature (2–8 °C). All this greatly reduced the manufacturing, storage and transportation cost. Therefore, we had designed a llama-human chimeric antibody which could act as a potential therapeutic agent that reduces LDL-c and total cholesterol. Further studies to confirm its potential role as a therapeutic agent are required.

## Conclusions

In conclusion, we discovered and designed a novel lama-human chimeric antibody and verified its function by in vitro and in vivo assays. Due to the high yield and low cost of *Pichia pastoris*, lipid-lowering effect and strong stability, the llama-human chimeric antibody (VHH-Fc) offers a potent therapeutic candidate for the control of the serum lipid level.

## Materials and methods

### Animal

One female llama (three years of age), raised in LvMeng Co., Ltd (Shenzhen, Guangdong, China) was used in this study. 500 μg emulsified hPCSK9 antigen (Sinobiological, China, Cat# 29698-H08H) with adjuvant was subcutaneously injected four times at monthly intervals. The isopyknic complete Freund’s adjuvant (500 μg, Sigma-Aldrich, USA, Cat# F5881-10ML) was used for the primary immunization, and incomplete Freund’s adjuvant (500 μg, Sigma-Aldrich, USA, Cat# F5506-10ML) was used for the other three immunizations. One month after the final immunization, 50 mL peripheral blood was collected, from which the mononuclear cells were isolated by adding ficoll-paque plus (GE Healthcare, USA, Cat# 45-001-749) for density gradient centrifugation. Forty-eight 4-weeks-old SPF male Sprague–Dawley (SD) rats were fed in the GemPharmatech Co., Ltd (Nanjing, Jiangsu, China) for pharmacodynamic studies.

### Phage display and kinetic screening

The total RNA was extracted using an RNEasy kit (Qiagen, Hilden, Germany, Cat# 74104), according to the manufacturer’s protocol. The immune library for phage display was then constructed and was found to be similar to previous reports [32]. Briefly, using hPCSK9-coated 96-well plates, four rounds of bio-panning were performed, with each round consisting of four steps - phage binding to the hPCSK9, washing, phage elution, and amplification. In the last step, the phages produced by panned individual clones were tested for their binding ability to the hPCSK9. It is a simple and convenient method to screen for clones before antibody purification. However, the best specific VHH clones cannot be correctly identified only by ELISA. To resolve this issue, we performed kinetic screening of the lysates by SPR (Biacore T200, GE Healthcare, USA) [35]. Due to low VHH levels in the lysate, high level antigen coupling is required. Therefore, hPCSK9-his tagged antigen (Sinobiological, China, Cat# 29698-H08H, 20 μg/mL in 10 mM sodium acetate (pH5.0, GE Healthcare, USA, Cat# BR-1003-51)) was captured on a CM5 chip (GE Healthcare, USA, Cat# 29104988) for 420 s. Finally, an average of 18000 RU of captured hPCSK9 antigen was immobilized on the flow cell 2, with flow cell 1 maintained as blank. The immobilization step was carried out at a flow rate of 30 μL/min in 1× PBS buffer. After lysis by TES buffer (0.2 M Tris–HCl pH 8.0, 0.5 mM EDTA, 0.5 M sucrose), the relative binding and stability values of each clone was measured by the SPR when these 22 supernatants flowed over the hPCSK9-coated CM5 chip. The result plot could be automatically generated by the built-in evaluation software between the stability value and the binding value. These values are positively correlated with the affinity for hPCSK9. The off rate and kinetics binding experiments were performed at 25 °C. The binding and dissociation time was set at 120 s and 140 s, respectively. The time points of the binding (at ~ 120 s) and stability values (at ~ 135 s) are system default settings. Glycine (pH 2.0, GE Healthcare, USA, Cat# BR-1003-55) was used as the regeneration buffer.

### Protein expression, purification and affinity determination

At first, VHHs’ expression was induced for these four recombinant *E. coli* by 0.1 M IPTG (Sangon Biotech, China, Cat# A600168-0025). To improve the expression and purification efficiency, the recombinant pPICZ-α vectors were constructed, transfected and integrated into the genome of the *Pichia pastoris* X33 (yeast) to express the VHH related proteins. Recombinant secretion expression was induced by adding 0.5% methanol every 24 h and shaking at 220 rpm at 30 °C for 5 days. The supernatants of the culture medium were harvested and assayed for recombinant protein expression. The supernatant was filtered with 0.22 μm PES filter membrane (Millipore, USA, Cat# GPWP02500). The 6his-tagged proteins were purified with the Histrap FF column (GE Healthcare, USA, Cat# 17-5319-01), and Fc-fused proteins were purified with the Protein A column (GE Healthcare, USA, Cat# 17040201).

SPR technology was used to detect the kinetics binding and dissociation process of the VHH proteins. Briefly, hPCSK9-his tagged antigen (20 μg/mL, in 10 mM sodium acetate, pH5.0. Sinobiological, China) was captured on the flow cell 4 of the CM5 chip (GE Healthcare, USA, Cat# 29104988) at ~ 800 response unit (RU), with flow cell 3 maintained as blank. All the binding assays were performed at 25 °C on the Biacore T200 instrument (GE Healthcare, USA). The binding and dissociation time was set at 120 s and 180 s respectively. A 6 or 7-points, double fold dilution of different molar concentrations of these VHH-based proteins were injected and the sensorgrams were globally fitted with a floating R_max_ using the built-in evaluation software. The regeneration buffer was glycine (pH 2.0, GE Healthcare, USA, Cat# BR-1003-55). The calculation formula of the affinity (K_D_) is as followings: K_D_ (nM) = k_off_ (1/s)/k_on_ (1/Ms). k_off_ is the dissociation constant, and k_on_ is the binding constant.

### Epitope binning test

First, the epitope binning assay was performed by the SPR at 25 °C. Briefly, the evolocumab (Amgen, USA, CAS# 1256937-27-5) was diluted in PBS (1 μg/mL) and captured on the flow cell 2 of the Protein A chip (GE healthcare, Cat# 29127555) at ~ 270RU, with flow cell 1 as blank. Next, the hPCSK9-his tagged antigen (5 μg/mL, in PBS) was injected into the dual flow cells as the Sample 1 for 90 s. All the evolocumab-specific epitopes on the hPCSK9 were occupied by the evolocumab and the Sample 1 (PCSK9-his)’s curve would reach a plateau. Then the VHH-6his antibodies (5 μg/mL, in PBS) were injected into the dual flow cells as the Sample 2 for 60 s. If these VHHs had different PCSK9 epitopes with evolocumab, the curve would continue to rise some response unit. If not, the curve would stay at a stable RU level. The dissociation time of the Sample 1/2 were set as 120 s. Similarly, the pH2.0 glycine (GE Healthcare, USA, Cat# BR-1003-55) was take as the regeneration buffer.

Furthermore, ELISA was also performed to verify the SPR results. The evolocumab (100 ng/well, Amgen, USA, CAS# 1256937-27-5) was coated on the ELISA plate overnight at 4 °C. After washing and 3% BSA (Sangon Biotech, China, Cat# A600332-0005) blocking, the hPCSK9-Fc tagged antigen (1 μg, Sinobiological, China, Cat# 29698-H05H) was added for one hour at 25 °C.After washing, the serial dilution VHH was added to bind the other epitopes on the hPCSK9 protein for one hour at 25 °C. Then horse radish peroxidase tagged anti-his antibody (1:5000 dilution, Abcam, UK, Cat# ab184607) was taken as the second antibody for one hour at 25 °C. After washing, 100 μL tetramethylbenzidine was added and the plate was incubated in the dark at 25 °C for 10 min (Abcam, UK, Cat# ab171522). The reaction was stopped by 50 μL 1 M sulfuric acid. The OD450 absorbance was measured by a microplate reader (Epoch, BioTek, USA).

### LDL-uptake assay

Huh7 and HepG2 cells were plated at a density of 5 × 10^5^ cells/well in 96-well plates (BIOLEGEND, USA, Cat# 423501) in Dulbecco’s modified eagle medium (DMEM, GIBCO, USA, Cat#11965084) supplemented with 10% fetal bovine serum (FBS, GIBCO, USA, 10099141). After 24 h, five groups were designed. The first group of cells were taken as the blank control (no addition of hPCSK9 and sdAbs). The second group of cells were taken as the negative control (addition of 0.080 μM hPCSK9 alone). 0.080 μM purified hPCSK9 and the other three groups of sdAb-based proteins (in three doses, 0.375 μM, 0.750 μM and 1.500 μM) were pre-incubated at 37 °C in the same medium and added onto the cells (three wells/condition). After 1 h at 37 °C, 10 μL of LDL-BODIPY (Invitrogen, USA, Cat# L3483) was added to the cell medium (6 μg/mL), and cells were further incubated for 3 h. After three washes in dulbecco’s phosphate buffered saline (DPBS, Gibco, USA, Cat# C14190500BT), plates were scanned on a Tecan Infinite M1000 PRO (Tecan, Switzerland). LDL-uptake level was measured in each well as an average fluorescence intensity.

### Pharmacodynamics

Pharmacodynamics was performed in the GemPharmatech Co., Ltd (Nanjing, Jiangsu, China). Briefly, the rats were divided into six groups (eight rats in each), of which there were four *hPCSK9* transgenic rat (Tg^+^ rat) groups and two normal control groups. The *hPCSK9* transgenic rats were produced through genomic random insertions by the injection of the *hPCSK9* gene into the fertilized eggs according to previous reports [13, 16, 30]. The successful transgenic rats were screened by PCR verification for the presence of the *hPCSK9* genes. These Tg^+^ rats were induced by a high-fat diet for 8 weeks. Of the four Tg^+^ group rats, three groups were, respectively injected in the tail vein with PBS, evolocumab, and B11-Fc (20 mg/kg). The remaining *hPCSK9* Tg^+^ group rats were fed with the normal diets. The group IDs as assigned were, Tg^+^_high fat_PBS, Tg^+^_high fat_Evolo, Tg^+^_high fat_B11-Fc and Tg^+^_normal diet. Two normal control groups were maintained. The first group was fed with the normal diets (ID: SD_normal diet group). The second group was fed with the high-fat diet and injected with the evolocumab (ID: SD_high fat_Evolo). The rats were injected at day 0 and 7 and sacrificed at day 20. Blood samples were collected and total cholesterol (CHOL) and liver LDL-c levels were determined by an automatic biochemical analyzer (Hitachi 7020, Japan).

### Stability test

Furthermore, we evaluated the storage stability and thermal stability of the B11-Fc antibody for its medicinal potentials by the affinity determination. Briefly, the freshly prepared B11-Fc protein (1 mg/mL in 1 × PBS) was divided into six groups. The first five groups were used for the storage stability evaluation. After the preservation at room temperature (~ 25 °C), the storage stability was studied by comparing their affinities at several points in time (at the end of 1st, 4th, 7th, 10th, and 13th weeks). The other group was used directly for the thermal reaction stability evaluation at 40 °C by setting the reaction temperature of the Biacore T200 (GE Healthcare, USA). The capture method was used to determinate the affinity based on Protein A chip (GE healthcare, Cat# 29127555). The B11-Fc protein (1 μg/mL, in PBS) was captured on the flow cell 2 at ~ 250RU, with flow cell 1 as blank. Next, the hPCSK9 antigen (100 nM, doubling dilution, Sinobiological, China, Cat# 29698-H08H) was injected into the dual flow cells for binding 180 s or 240 s. Similarly, the pH2.0 glycine (GE Healthcare, USA, Cat# BR-1003-55) was take as the regeneration buffer.

### Statistics

All the statistical analysis was performed with the software Graph-Pad PRISM 5. All the data represented is an average value of at least three independent experiments (± S. E). Two-tailed Student’s t-test was performed to assess the statistical significance of the data sets. *P* < 0.05 was considered as statistically significant (*P < 0.05, **P < 0.01, ***P < 0.001, ns: not significant).

## Supplementary information

**Additional file 1: Fig. S1.** The LDL-c metabolism mechanisms and the effects of the PCSK9 protein. Under normal circumstances, LDL-c in the serum binds to the LDLR on the liver cell surface and then be be degraded by lysosome. LDLR would recover its activity on the cell surface (the pathway of orange arrows). PCSK9 protein plays a vital role in cholesterol homeostasis by binding to the LDLR. High level PCSK9 competitively binds LDLR with LDL-c, which would cause disorder of LDL-c metabolism. The LDLR would be degraded abnormally intracellularly (the pathway of green arrows). The antibodies (Evolocumab in red or VHH-Fcin blue) can bind the PCSK9, promote LDLR recovery and restore the LDL-c metabolism at some level.

**Additional file 2: Fig.S2.** The structure schematic diagram and the amino acid sequence of the PCSK9 protein. The PCSK9 protein is composed of signal peptide (amino acid NO. 1-30), pro-domain (NO. 31-152), catalytic domain (NO. 153-425) and C-terminal domain (NO. 426-692). It consists of 692 amino acid residues. The sequence of the PCSK9 protein was shown every 60 amino acid residues in a row.

**Additional file 3: Fig. S3.** The serological antibody titer test of the immunized llama. The horizontal axis represents five dilution concentrations of the llama serum. The vertical axis represents the OD450 value. ‘(+)’ and ‘(−)’refer to the coating and no-coating of the antigen hPCSK9 to ELISA plates. ‘Pre’ refers to the collected serum before the immunization. ‘Post’ refers to the collected serum 1 month after last immunization. ‘Blank’ refers to the PBS control of the ELISA assay. The star (#) represents serology positive (the OD450 ratio of post-immune serum/pre-immune serum ≥ 2.1).

**Additional file 4: Table S1.** The sequences of the sdAbs.

**Additional file 5: Fig. S4.** The stability test by the affinity determination. (A-E) The storage stability test was performed by the affinity determination of the B11-Fc preserved for 1, 4, 7, 10 and 13 weeks. (F) The thermal stability test was performed by the affinity determination of the B11-Fc at 40 °C reaction temperature. Each colored line represents one antibody concentration. The black lines represent the automatic fitting curves by the built-in evaluation software. The binding and dissociation time was set at 180 s/240 s and 240 s respectively, and the protein injection time point was automatically set as 0 s by the built-in evaluation software.

## Data Availability

Not applicable.

## Abbreviations

CHOL: Total cholesterol
CVD: Cardiovascular diseases
D-PBS: Dulbecco’s phosphate buffered saline
DMEM: Dulbecco’s modified eagle medium
ELISA: Enzyme-linked immunosorbent assay
Fc: The constant region fragment of the human immunoglobulin gamma
hPCSK9: Human proprotein convertase subtilisin/kexin type 9
HepG2: Human hepatoma cells G2
Huh7: Human hepatoma cells huh7
IgG: Immunoglobulin gamma
IPTG: Isopropyl-β-d-thiogalactoside
LDL: Low density lipoprotein
LDL-c: Low density lipoprotein cholesterol
LDLR: Low density lipoprotein receptor
OD450: The optical density value at 450 nm
PCSK9: Proprotein convertase subtilisin/kexin type 9
RFU: The relative fluorescence unit
RU: Response units
SD rat: Sprague–Dawley rat
sdAb: Single domain antibody
SPR: Surface plasmon resonance
TES buffer: 0.2 M Tris–HCl pH 8.0, 0.5 mM EDTA, 0.5 M sucrose
Tg+: *hPCSK9* transgenic rats
VHH: High variable region of the heavy chain antibody
kDa: Kilo dalton
PBST: Phosphate buffer solution with 0.05% tween 20
RNA: Ribonucleic acid
