# Supplementary figures and images for: The novel llama-human chimeric antibody has potent effect in lowering LDL-c levels in hPCSK9 transgenic rats
Source: Clin Transl Med. 2020 Feb 13;9:16. doi: 10.1186/s40169-020-0265-2 (PMC7018876; doi:10.1186/s40169-020-0265-2)

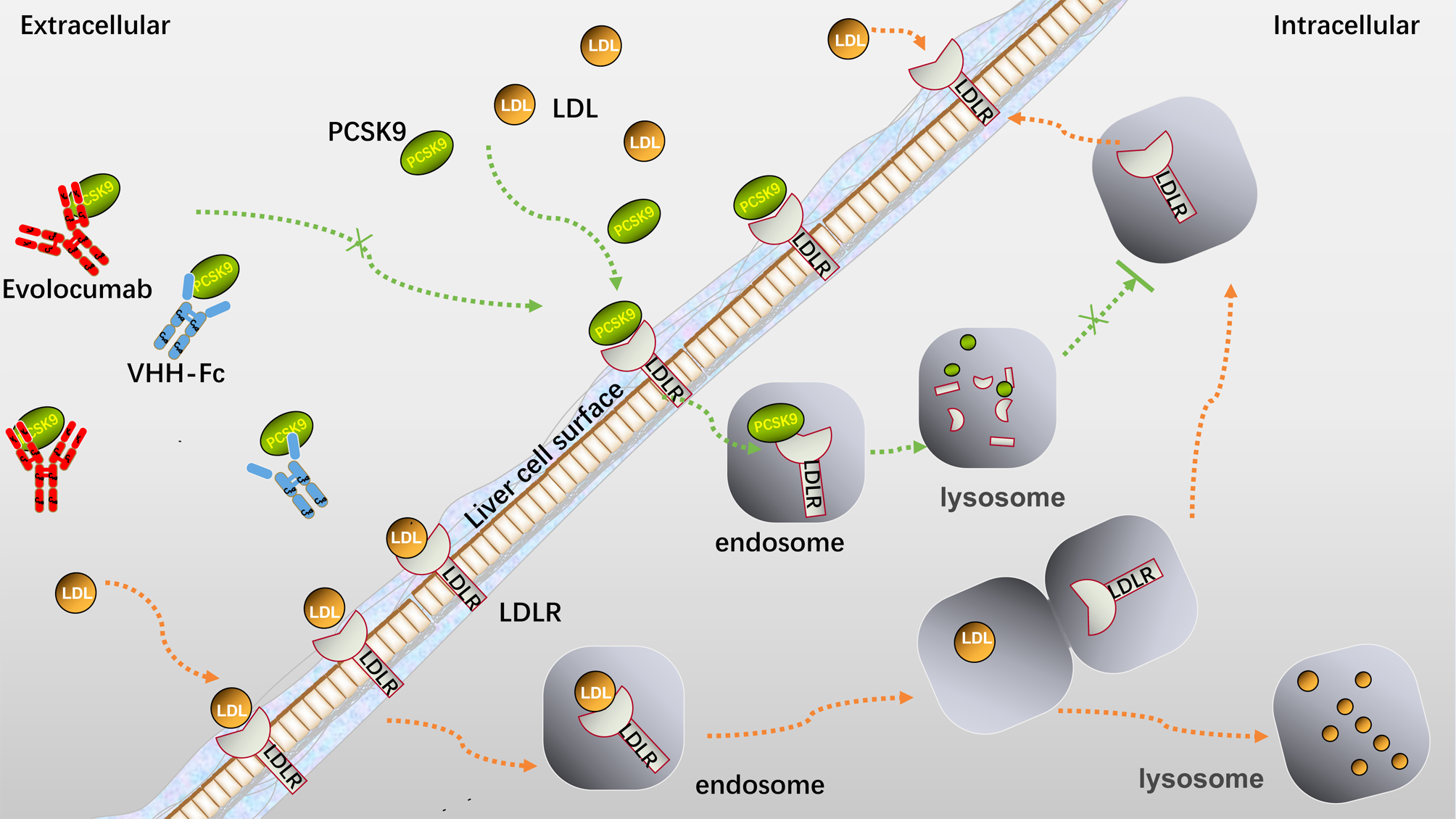

Supplement: Supplementary file 1 — Additional file 1: Fig. S1. The LDL-c metabolism mechanisms and the effects of the PCSK9 protein. Under normal circumstances, LDL-c in the serum binds to the LDLR on the liver cell surface and then be be degraded by lysosome. LDLR would recover its activity on the cell surface (the pathway of orange arrows). PCSK9 protein plays a vital role in cholesterol homeostasis by binding to the LDLR. High level PCSK9 competitively binds LDLR with LDL-c, which would cause disorder of LDL-c metabolism. The LDLR would be degraded abnormally intracellularly (the pathway of green arrows). The antibodies (Evolocumab in red or VHH-Fcin blue) can bind the PCSK9, promote LDLR recovery and restore the LDL-c metabolism at some level. [file 40169_2020_265_MOESM1_ESM.tif]

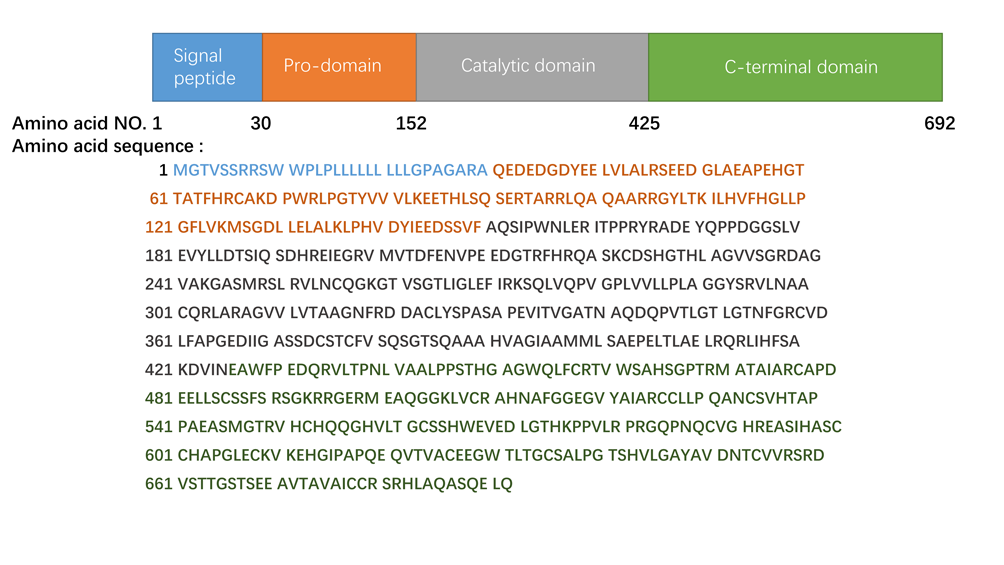

Supplement: Supplementary file 2 — Additional file 2: Fig.S2. The structure schematic diagram and the amino acid sequence of the PCSK9 protein. The PCSK9 protein is composed of signal peptide (amino acid NO. 1-30), pro-domain (NO. 31-152), catalytic domain (NO. 153-425) and C-terminal domain (NO. 426-692). It consists of 692 amino acid residues. The sequence of the PCSK9 protein was shown every 60 amino acid residues in a row. [file 40169_2020_265_MOESM2_ESM.tif]

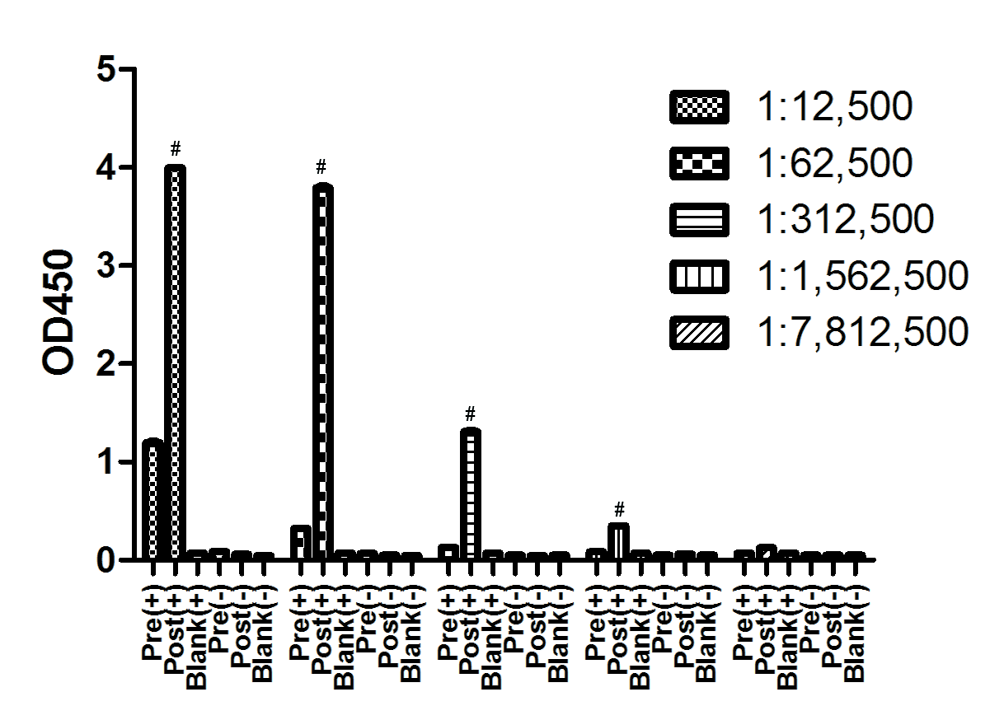

Supplement: Supplementary file 3 — Additional file 3: Fig. S3. The serological antibody titer test of the immunized llama. The horizontal axis represents five dilution concentrations of the llama serum. The vertical axis represents the OD450 value. ‘(+)’ and ‘(−)’refer to the coating and no-coating of the antigen hPCSK9 to ELISA plates. ‘Pre’ refers to the collected serum before the immunization. ‘Post’ refers to the collected serum 1 month after last immunization. ‘Blank’ refers to the PBS control of the ELISA assay. The star (#) represents serology positive (the OD450 ratio of post-immune serum/pre-immune serum ≥ 2.1). [file 40169_2020_265_MOESM3_ESM.tif]

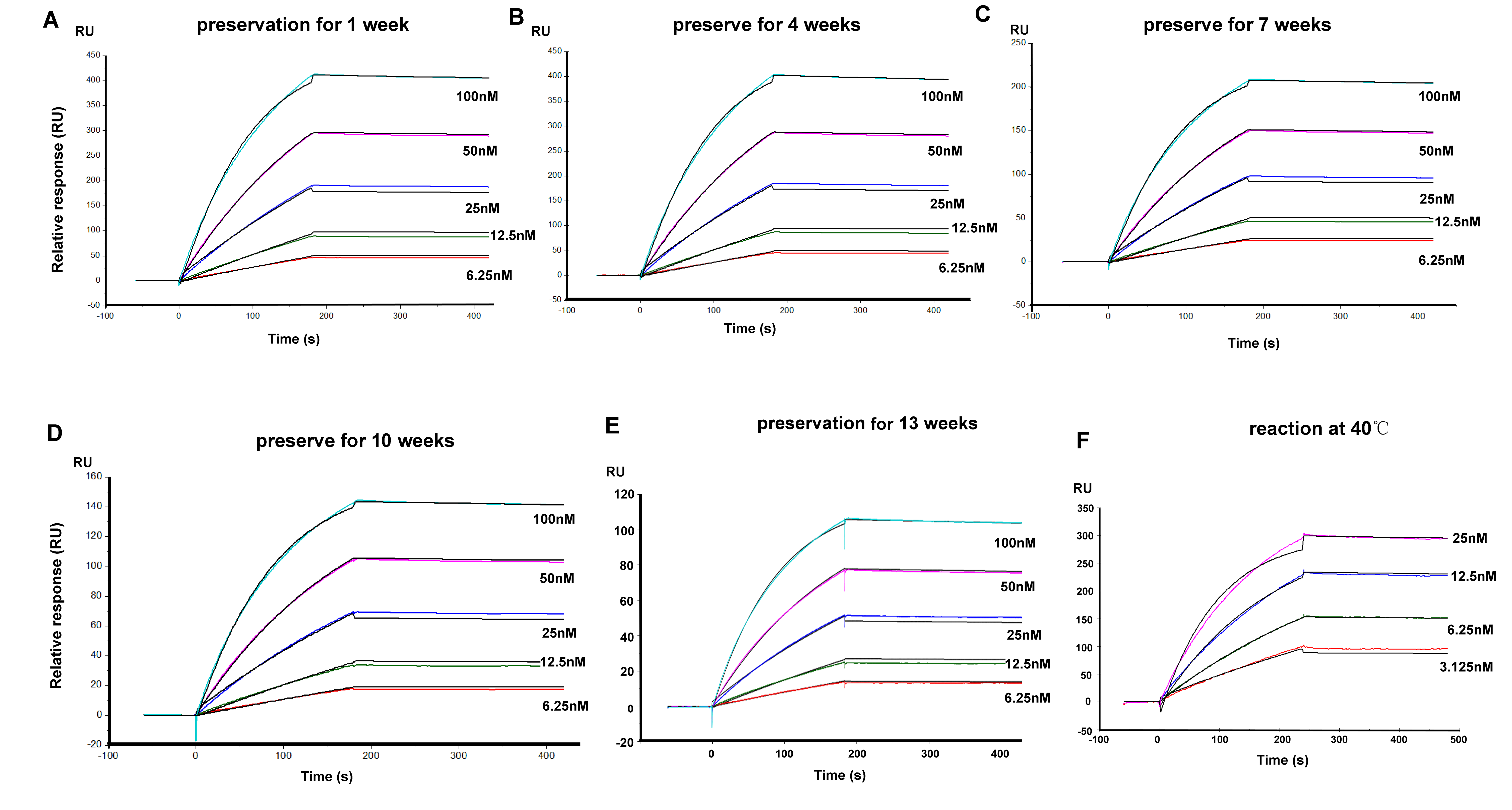

Supplement: Supplementary file 5 — Additional file 5: Fig. S4. The stability test by the affinity determination. (A-E) The storage stability test was performed by the affinity determination of the B11-Fc preserved for 1, 4, 7, 10 and 13 weeks. (F) The thermal stability test was performed by the affinity determination of the B11-Fc at 40 °C reaction temperature. Each colored line represents one antibody concentration. The black lines represent the automatic fitting curves by the built-in evaluation software. The binding and dissociation time was set at 180 s/240 s and 240 s respectively, and the protein injection time point was automatically set as 0 s by the built-in evaluation software. [file 40169_2020_265_MOESM5_ESM.tif]
