# Supplementary material for: The novel llama-human chimeric antibody has potent effect in lowering LDL-c levels in hPCSK9 transgenic rats
Source: Clin Transl Med. 2020 Feb 13;9:16. doi: 10.1186/s40169-020-0265-2 (PMC7018876; doi:10.1186/s40169-020-0265-2)
Supplement: Supplementary file 4 — Additional file 4: Table S1. The sequences of the sdAbs. [file 40169_2020_265_MOESM4_ESM.docx]

**Table S1** The sequences of the sdAbs

| sdAb ID | Sequences (FR1-CDR1-FR2-CDR2-FR3-CDR3-FR4) | IGHV | IGHJ |
| --- | --- | --- | --- |
| VHH-B11 | EVQLVESGGGSVQAGGSLRLSCTVS-GYTYSSNC-MGWFRQAPGKEHEGVAS-IYIGGGST-YYADSVKGRFTISQDNAKNTVYLQMNSLKPEDTAMYYC-AVGCQGLVDFGY-WDQGTQVTVSS | IGHV3S40 | IGHJ6 |
| VHH-H12 | GGGLVQAGGSLRLSCAAS-RSTFSGYA-MAWFRQAPGKEREFVAC-IEREIPGHPAWSGLT-YYADSKKGRFTISRDNAKNTVYLQMNSLKPEDTAVYYC-AAGLKYPAQKHYDYDY-WGQGTQVTVPS | IGHV3S56 | IGHJ4 |
| VHH-A6 | PGAAAGVGGGSVQAGGSLRLSCAAS-RYTDRTRC-IAWFRQVPGKEREGVAC-LDRAGGQS-AYADSAKGRFTVSQDNAGNTVYLQMDNLIPEDSAMYYC-AAAGVGQWYTCLQKFIRDKRSFAN-WGQGTQVTVSS | IGHV3S9 | IGHJ4 |
| VHH-G8 | DVQLVESGGGSVQAGGSLTLSCVVS-GYRGQKIC-TGWFRQFPGMEREAVAR-ILPRGPNT-QYTDSVKGRFTISQDAAKNTVNLQMSSLKPKDTAMYY–CAQGWGGASDWALQPRRYNY-WGQGTQVTVSS | IGHV3S60 | IGHJ4 |

sdAb ID represents the single domain antibody. Sequences (FR1-CDR1-FR2-CDR2-FR3-CDR3-FR4) represents the amino acid sequences of each sdAb from ‘FR1’ to ‘FR4’. FR and CDR represent the framework and complementary determining regions of the variable region of heavy chain antibody. The IGHV and IGHJ represent the V and J gene usages of the sdAb sequences when aligned to the *Vicugna pacos* germline genes from IMGT-VQUEST (http://www.imgt.org/IMGT_vquest/input). *e.g.* IGHV3S40 represents the S40 subfamily gene of the clan V3 family of the *Vicugna pacos* IGH germline.
